# Understanding the crisis in harm reduction funding in Central and Eastern Europe

**DOI:** 10.1186/s12954-020-00428-6

**Published:** 2020-10-22

**Authors:** Michal Miovský, Silvia Miklíková, Viktor Mravčík, Jean-Paul Grund, Tereza Černíková

**Affiliations:** 1 Department of Addictology, First Faculty of Medicine, and General University Hospital in Prague, Charles University, Prague, Czech Republic; 2 National Monitoring Centre for Drugs and Addiction, Office of the Government of the Czech Republic, Prague, Czech Republic; 3 CVO – Addiction Research Centre, Utrecht, The Netherlands

**Keywords:** Harm reduction, Drug policy, Comparative qualitative analysis, Financial support, System analysis, Case studies, Czech Republic, Slovak Republic

## Abstract

**Background:**

The harm reduction (HR) approach to injecting drug use was rapidly adopted in Central Europe following the fall of the Iron Curtain. The associated social and economic transformation had significant consequences for drug policies in the region. A large number of emerging services have been dependent on funding from a wide range of national and/or local funding programmes, which continue to be unstable, and closely associated with political decisions and insufficient institution building. A sharp distinction is made between health and social services, often without regard to client input. The main objective of the paper is to identify the causes of the funding problems currently faced by HR services in the context of their history of institution building which represents a major threat to the future of HR services in the region.

**Methods:**

Qualitative content analysis of documents was conducted in the development of two case studies of the Czech and Slovak Republics. The body of documentation under study comprised policy documents, including National Drug Strategies, Action Plans, ministerial documents, and official budgets and financial schedules, as well as documents from the grey literature and expert opinions.

**Results:**

The insufficient investments in finalising the process of the institution building of HR services have resulted in a direct threat to their sustainability. An unbalanced inclination to the institutionalisation of HR within the domain of social services has led to a misperception of their integrity, as well as to their funding and long-term sustainability being endangered. In addition, this tendency has had a negative impact on the process of the institutionalisation of HR within the system of healthcare.

**Conclusion:**

The case study revealed a lack of systemic grounding of HR services as interdisciplinary health-social services. The aftermath of the financial crisis in 2008 fully revealed the limitations of the funding system established ad hoc in the 1990s, which remains present until today, together with all its weak points. The entire situation is responsible for the dangerous erosion of the interpretation of the concept of harm reduction, which is supported by various stereotypes and false, or ideological, interpretations of the concept.

## Background

The history of harm reduction policies concerned with drug-related problems in Western Europe can be traced back to as early as the first half of the 1970s [1], while in Australia and in the USA the first signs of the HIV epidemics spurred the first needle exchanges and subsequent harm reduction efforts. Initially, harm reduction advocates faced an uphill battle but in the European Union the hill has become much less steep over the years, with “consensus replacing controversy” [2]. In 2018, there were 335 needle and syringe programmes in the USA, organised by a rather well-developed grassroots harm reduction movement [3–5]. The result of the federal government changing its position on needle and syringe programmes, leading to a partial repeal of the ban on federal funding for this service, is a recent increase in needle and syringe programmes in the USA. Although the use of federal funds to purchase sterile needles or syringes remains prohibited, federal funds are permitted to be allocated to other aspects of programmes that are needed to keep them in operation. While not completely without controversy, in Australia and the EU harm reduction has largely been mainstreamed, while it has gained a steadily growing foothold in Latin America and many Asian countries. In 2018, 86 countries worldwide had implemented some form of needle and syringe programme and 86 countries had implemented opioid substitution treatment [3].

The harm reduction (HR) approach to drug use was rapidly adopted in Central Europe (CEE) following the fall of the Iron Curtain. The enormous speed of the social and economic transformation, marked by little experience with an open market, had significant consequences for the drug policy. A large number of newly emerging services were (and often still are) dependent on funding through a wide range of central and/or local programmes of subsidies, the granting of which was arbitrary and followed no rigorous structure. Since the beginning, such a system has been fragile and unstable, particularly because of its inseparable association with political decisions and insufficient institutionalisation. In this respect, it is characteristic of the former Soviet bloc that a division is made, often artificially and without regard to context, between health and social services, although this may impair their interrelationship. This phenomenon seems to have become the greatest danger to the emerging network of HR services, which can be demonstrated in their development in the Czech and Slovak Republics pointing out the major shortcomings of the system.

The aim of this paper is to conduct analyses of the system of the provision of HR services in the context of the national drug policy vision (using case studies from the Czech and Slovak Republics) with special attention being focused on the way these services are founded and funded, as well as on the process of their institutionalisation. The main objective is to identify the causes of the current problems, which pose a danger to the configuration, definition, existence, and sustainability of these services. We also seek to establish whether this may indicate a structural problem which may need to be addressed generally, and not only in relation to the two countries under analysis.

## Harm reduction: from an emerging Public Health paradigm to a comprehensive set of interventions

“‘Harm Reduction’ refers to policies, programmes, and practices that aim primarily to reduce the adverse health, social, and economic consequences of the use of legal and illegal psychoactive drugs without necessarily reducing drug consumption. Harm reduction benefits people who use drugs, their families and the community. The harm reduction approach to drugs is based on a strong commitment to public health and human rights” [6]. The harm reduction approach entered the arena of drug policy, prevention, and treatment in the 1980s, when many countries experienced the onset of the HIV epidemic. Against the background of widespread HIV/AIDS infection among people who inject drugs (PWID) in the late 1980s, an increasing number of countries concluded that the “spread of HIV is a greater danger to individual and public health than drug misuse” [7]. That same period witnessed the rise of the New Public Health paradigm, reflecting a shift from a narrow biomedical perspective on disease (in this case, addiction) to a broader concept of health that recognises that health consequences (for example, those of drug use) are mediated and affected by specific environmental and situational factors, as well as the broader social framework [8–10].

Globally, harm reduction is becoming the dominant policy response to problem drug use and the associated harms, such as HIV infection. At this point, harm reduction is advocated by virtually all the major relevant United Nations organisations, including the WHO, UNAIDS, and others [11]. The best-known examples of harm reduction are, beyond a doubt, the provision of sterile injecting equipment to people who inject drugs and the provision of opioid substitution treatment. However, the term “harm reduction” refers to a much larger set of interventions. In the context of HIV prevention, treatment, and care for PWID, the WHO, UNODC, and UNAIDS have included nine interventions in what has been termed the “*essential package of interventions for effective HIV prevention and care for injecting drug users*” [12], which also includes resting, ART, the prevention of STIs, condom provision, information provision, and the prevention, detection, and treatment of viral hepatitis and TB.

Nonetheless, HIV or infectious diseases are not the only focus of harm reduction interventions and many programmes also target their efforts on other aspects of drug-related harm, addressing these on both the individual and community levels. For example, nine European countries (Belgium, Denmark, Germany, Luxemburg, Norway, Spain, Switzerland, and the Netherlands) have established drug consumption rooms in response to open drug scenes and public drug use. Additionally, Ireland and Portugal were expected to open, or did open, new facilities in 2019 [13]. In all of these countries, the implementation of these facilities was justified by both public health and public order considerations. Likewise, opioid overdoses are the subject of increasing concern in the European Union [14] and, elsewhere, an issue that is enhanced by synthetic opioids that pose a serious threat to individual and public health. In the USA and Canada, the current opioid epidemic is being driven by the use of synthetic opioids, and concerns also exist in Europe. Synthetic opioids that are usually used as medicines appear to be playing an increasing role in the drug problem in many parts of Europe. Additionally, the population of high-risk drug consumers is ageing, which, among other things, poses them an increased risk of overdoses. Among drug-related deaths in the EU, the proportion aged above 40 years increased from around one in three in 2006 to nearly one in two in 2015 [15]. Pioneered in the USA, overdose prevention strategies, including naloxone distribution [16], are increasingly being offered by European harm reduction programmes as well. In 2018, take-home naloxone programmes were implemented in 12 European countries [17]. In many countries, harm reduction programmes have developed specific approaches for reducing the harms associated with stimulant drugs, addressing medical as well as psychosocial problems [18]. Rhodes and Hedrich [2] referred to harm reduction as a “combination intervention”, merging approaches from many different disciplines and involving various stakeholders in designing pragmatic and tailored interventions for drug-related problems. More recently, increasing attention has been paid to the complex relationships between drug legislation and enforcement, harm reduction and public health, and human rights (REFs). Indeed, since its inception halfway through the 1980s, the concept of harm reduction and its implementation has continued to develop and venture into new areas of policy and service provision. The harm reduction approach is increasingly being applied to the use and problems associated with other drugs (e.g. crack, alcohol, or party drugs) and to non-drug problems (such as teenage pregnancy, domestic violence or juvenile offending, and poverty and food banks). In this process, an extremely wide (and expanding) range of disciplines and professions has collaborated in developing innovative and pragmatic interventions and policies, which transcend the specific outlook or interests of their particular professions or disciplines. In doing so, they have contributed to creating and defining what could be termed a *Trans-Disciplinary Bricolage* of synergetic practice, policy, empirical research, and theory.

Research suggests an association between the availability and coverage of harm reduction programmes and the spread of HIV infection. Countries providing efficient and accessible harm reduction services, needle exchange programmes, and opiate substitution treatment in particular have been effective in reducing the incidence of HIV among PWID [e.g. 19]. Opiate substitution treatment may also reduce the incidence of HCV [20], while “full participation” in combined opiate substitution and exchange programmes results in more effective reductions in HIV and HCV transmission than participation in “stand-alone” programmes [21]. A review of harm reduction interventions among PWID found promising results pertaining to a range of other health consequences, although it highlighted the “uneven presence of high-quality review evidence” [22].

Beyond legitimate concerns over the HIV epidemic and its potential consequences and the absence of a sufficient response of traditional (abstinence-oriented) drug services, a large part of the acceptance of harm reduction can be credited to: (i) its contributions in controlling HIV epidemics among PWID around the globe; (ii) the early support provided by a number of key international civil society players (such as the Open Society Institute of the philanthropist George Soros); (iii) the development of strategic alliances of practitioners, researchers, activists, and policy makers, resulting in an extensive “Best Practice” and “Evidence Base” in support of harm reduction interventions [e.g. 23]; and, finally, (iv) the support of significant UN bodies, such as the WHO, and major donors, such as the Global Fund. Harm reduction has clearly become a mainstream approach to problem drug use and drug-related harms, such as HIV infection [11].

The funding schemes influence the local interpretation of the concept of harm reduction and its implementation and the definition of its targets and goals, as well as the professional profile of those involved in providing harm reduction services. Moreover, without the inclusion of harm reduction into regular health and social care provision, the national or rather governmental ownership of harm reduction programmes cannot be achieved. This may in particular affect harm reduction (and HIV prevention) efforts in Central and Eastern Europe, where, despite important accomplishments in direct service provision, harm reduction policies remain immature and may be difficult to sustain.

During the last century, Central Europe was subject to rather specific developments which are without parallel in Western Europe or the USA. Likewise, culturally, historically, and politically, these developments differ from those in Russia or the majority of other post-Soviet countries outside Europe. These countries—Poland, the Czech Republic, Slovakia, and Hungary—are closely linked to the western part of Europe geographically, culturally, and historically. But the Iron Curtain separated these countries from Western Europe for about half a century. This had, for example, important consequences for the type and nature of substance use before the political transition. As closed borders prevented the import of “Western” drugs, such as heroin or cocaine, people who used drugs in all four countries and throughout most of the former Soviet Union turned to crude homemade injectable opioids or stimulants [24, 25]. Poland was the only Central European country to experience an HIV epidemic among people injecting home-made opiates, as early as in the late 1980s, leading to the first needle exchange programmes in the country [26]. To this day, the remaining three countries have reported only sporadic cases of HIV infection among PWID. Nevertheless, the degree of implementation of syringe exchange programmes in the Central European countries varies and corresponds to neither their historical experience with HIV among PWID within their territories, nor the current epidemiological situation.^1^

There was no system of public health services available to PWID during the Communist era; injecting use was a taboo and open drug scenes did not exist prior to the late 1980s [24, 29–31]. After the political changes and the resulting liberalisation of trade and travel restrictions, these four countries found themselves confronted with a “drug boom” in the 1990s and had to develop services for people who use drugs at a fast pace. They can be viewed as “laboratories” in which drug use and interventions for people who use drugs have developed more rapidly than in Western Europe, in particular in the domain of harm reduction.

## Methods

Analytical work with these qualitative data was mainly based on three techniques (procedures) [32]: (i) capturing patterns, themes, or “gestalts” in the direct transcriptions of interviews; (ii) linking sub-elements to general categories; and (iii) marking and interpreting relationships between qualitative variables. The diversity and non-homogeneity of the data sources required the consistent enforcement of data validity checking techniques. Two types of triangulation were used—of data sources and of methods, as well as the technique of contradictions [33, 34], which we used in “moderated team discussions”. Contradictory statements were discussed using an “indictment-accusation” method. (Each team member was asked to stand for and argue in favour of an appropriate extreme version of a statement.)

For the content analysis, field notes, official reports (National Focal Points in REITOX), strategies and ministry documents, historical documentation from private archives, shadow literature, and officially published papers and books were used.

## Results

### Czech–Slovak commonalities in developing harm reduction

The Federation of the Czech and Slovak Republics ceased to exist in 1993. Until then, both countries had shared a 75-year-long history since 1918, interrupted by the Second World War, when the Czech Republic became the German Protectorate of Bohemia and Moravia and Slovakia was a nominally independent state. There were some similarities before 1993 (e.g. joint legislation, the same strategies, etc.) and some differences (e.g. industry, people living in cities, etc.).

In the Czech Republic and Slovakia, harm reduction programmes developed against the background of the complex nationwide processes and events that marked the transformation period of the 1990s. These are likely to have significantly influenced and shaped the opportunities for the incorporation of such services into the system of the drug policy, as well as into the more general health and social policies pursued on the national level, and the limitations of such incorporation. In the light of the development of both countries’ respective drug scenes, drug services networks, and monitoring systems, the following characteristics stand out:

- HR services emerged and developed in a relatively favourable epidemiological situation as far as HIV/AIDS and viral hepatitis are concerned.

Both countries have shown a relatively positive epidemiological situation in terms of HIV/AIDS and hepatitis among both the general population and PWID.^2^ This had a crucial impact on the shaping of the content, form, and intensity of the public discussion on the drug policy and the funding of drug services, which are a delicate topic, particularly as regards harm reduction. The political dimension, i.e. the urgency with which both countries responded to the epidemiological situation and trends, was of major importance. While both countries have faced largely the same favourable epidemiological situation in terms of, for example, HIV or HCV incidence, harm reduction programming has received different political and financial support in both countries.

b Harm reduction interventions introduced in both countries showed a minor or declining emphasis on public health concerns, while a growing emphasis was placed on the social context and dimension of the services provided.

It is noteworthy that despite the relatively favourable epidemiological situation, in both countries HR services began to develop successfully, and the Czech Republic even built a relatively dense, accessible, and efficient network of services providing sufficient coverage of its entire territory. For the characteristics of harm reduction programmes in both countries, it is important to note that they are mainly provided by non-governmental organisations. The position of NGOs in this region is challenging; they often operate in a vague and inconsistent policy environment and play only a marginal role in governance processes [55, 56]. However, the development of this network was made possible mainly thanks to the emergence and growth of specific drug services. The involvement of the existing network of public health agencies (public health institutes and services) was negligible. In fact, the system of public health services stood aside from the process and has provided no structural support to people who use drugs up to this day. On the other hand, harm reduction services featured a strong social dimension and relationship with the social welfare network from the beginning, and these are being further enhanced even now. There may be various reasons for this:

c HR services were represented by no specific group of professionals, and the absence of involvement on the part of health professionals was particularly significant. Accordingly, the health sector has not provided much support for harm reduction services. Thus, they are often viewed as social or social rehabilitation services rather than public health services. This applies despite the fact that, in the Czech Republic, HR services are defined by the healthcare-related Act No. 65/2017 Coll., on the protection of health from the harmful effects of drugs (Section 27, which also provides a basic definition of professional care in terms of dependency), and in Slovakia they are referred to in the Bulletin of the Ministry of Health [57], which defines outreach work.
d The pressure posed by a rapid and hard-to-control increase in infectious diseases associated with injecting drug use was absent in these countries (see above).
e The systems of funding have been based on multiple sources. In the Czech Republic, for example, the subsidy system administered by the former National Drug Commission, presently the Government Council for Drug Policy Coordination (GCDPC)—Government Office, existed in parallel with the subsidy schemes of the Ministry of Labour and Social Affairs (MoLSA) and of the Ministry of Health (MoH). In Slovakia, additionally, long-term financial support was provided by Nadácie otvorenej spoločnosti—Open Society Foundation (OSF), which played a significant role in the context of the funding of HR services in that country (see below).
f In both countries, the systems of social care developed in line with their respective strategies. These processes involved the integration of harm reduction programmes into the systems of social services, specifically social prevention services, primarily in the form of outreach programmes and drop-in centres, two out of a total of 32 types of social services defined in the Czech Republic by Act. No. 108/2006 Coll. on social services. As a result, the system of the provision of social services was newly provided with a strong legal framework, which also serves as the basis for funding. While the portfolio of social affairs has provided standard coverage for what is denominated and categorised as a social service, the health departments in both countries have traditionally, and for a long time, adopted very reserved and recently almost negative standpoints on the integration of harm reduction programmes into health policies, as well as on the provision of financial support for such programmes; they are simply viewed as not health/medical enough and inconsistent with the notion of “healthcare”. Despite their obvious public health dimension, harm reduction programmes thus continue to be increasingly dependent on non-health resources, which make their integration into the system of health services even more difficult.
g The association of illicit drugs with pre-1989 political dissent [29] may have led to a deep-rooted and enduring idea of drugs being related to the disruption of society and posing an immediate danger to it. Although transformed, this notion still seems to echo in the present. This leads to the stigmatisation of PWUD and drug services, primarily harm reduction services, which can be demonstrated by a number of community and political measures against drug services (Table 2).
h The discussion about the effectiveness and development of harm reduction programmes often bears signs of populism, lacks relevant arguments, and abounds in simplifying judgements that are in sharp contrast with the state of the art of research and the results of studies concerned with this area. Even members of the professional community are frequently heard to make statements to the media that contradict the evidence-based approach. To a great extent, this may result from a lack of awareness and understanding on the part of certain professional groups (including psychiatrists and clinical psychologists, in particular) and, to some degree, from the persistent residue of what was called the “narcological system of service management” that was typical of the countries of the former Soviet bloc. Ideological arguments and legislative systems framing drug use and drug possession as a crime complicate the implementation of a full range of harm reduction interventions, including heroin-assisted programmes, drug consumption facilities, or harm reduction interventions in prisons.
i Another significant factor is the insufficient pressure exerted by civil society. These countries show an obvious lack of the voices of people who use drugs and their parents and partners, drug services, and communities in policymaking and in mass media reporting.

### Czech–Slovak differences in developing harm reduction

The first HR services for people who inject drugs were established in the Czech Republic in the early 1990s. The programmes that came into existence at that time were later used as models for the emerging network. The concept of the provision of such services was adopted from abroad (see, for example, the design of the first Drug Policy Strategy [58]) and was also closely linked to efforts seeking to monitor the development of the situation^3^ and to have the opportunity to subject it to further research activities. Thanks to the structural support provided on the national level, which culminated between 1997 and 2001, the Czech Republic managed to build what, in the European context, may be considered a dense network of relatively easily accessible and efficient harm reduction programmes [35]. For example, the numbers of needles and syringes exchanged have been rising in recent years (Table 1) and complex issues, such as collaborating with pharmacies and reinforcing the network of HR programmes by their involvement in the programmes, have been opened.^4^ Additional innovative interventions, including the distribution of gelatine capsules intended primarily for people who use pervitin as an oral alternative to riskier injecting practices, have also been developed [60, 61].

**Table 1:** Overview of development in selected indicators in the Czech Republic (2005–2018)

Indicator	2005	2006	2007	2008	2009	2010	2011	2012	2013	2014	2015	2016	2017	2018
Total number of newly identified HIV cases in the Czech Republic	90	91	121	148	156	180	153	212	235	232	266	286	254	208
Including the number of cases who may have contracted the infection through injecting drug use*	5	5	17	12	7	7	12	10	10	15	11	11	8	11
Needle and syringe programmes (NSPs)—number of programmes in the Czech Republic	88	93	107	98	95	96	99	103	110	105	104	104	108	107
Number of needles and syringes distributed in the Czech Republic (in thousands)	3272	3869	4457	4644	4859	4943	5293	5356	6175	6594	6403	6469	6402	6932

^*^Including the mixed category of PWID/MSM, where both risk factors are present

Source: [35, 37]

The situation in Slovakia was somewhat different. Unlike in the Czech Republic, the resources available to the Slovak counterpart of an interdepartmental funding scheme, called the Anti-drug Fund, have never been used to provide structural support for HR services and thus create conditions for their development. On the contrary, the attempt to open discussion on a quest for a structural solution (integrating the public health perspective) by developing uniform quality standards for low-threshold services [62] was rejected by the health sector at the very beginning for reasons which are difficult to understand [63]. While compromised by numerous major shortcomings, this initiative, the highlights of which were supported by the Bulletin of the Ministry of Health [57] published five years earlier, was promising in terms of the further development, strengthening, and stabilisation of the health component of HR services within the Slovak healthcare system. It should be noted that the key argument used to support the negative position was the senseless (although symbolic and essential with a view to the subject matter of this paper) requirement that the HR programmes should restrict their activities to the domain of social interventions only^5^ and give up the ambition of conducting public health interventions. On the other hand, financial support from the OSF in the years 1998–2005 made it possible to establish a small network of services in the largest Slovak cities which continued to be maintained and developed until this source of funding was disengaged. Unfortunately, HR services have not been securely integrated into the financial framework of social and health services in the Slovak Republic (Table 2).

**Table 2:** Overview of development in selected indicators in the Slovak Republic (2005–2018)

Indicator	2005	2006	2007	2008	2009	2010	2011	2012	2013	2014	2015	2016	2017	2018
Total number of newly identified HIV cases in the Slovak Republic	21	27	39	49	45	25	46	43	80	83	79	82	66	82
Including the number of cases who may have contracted the infection through injecting drug use	0	1	1	3	1	1	0	1	1	1	3	1	0	1
Needle and syringe programmes (NSPs)—number of programmes in the Slovak Republic*	10	13	14	9	n.a	7	9	9	8	8	8	8	8	n.a
Number of needles and syringes distributed in Slovakia (in thousands)	362	384	421	254	318	317	352	350**	321	275	347	358	396	n.a

^*^The first exchange programme in Slovakia was launched in 1994 [26]

^**^Estimate from the annual reports of HR organisations

Sources: [38–54]

Both the pieces of legislation pertaining to the Ministry of Labour and Social Affairs and the Ministry of Health, respectively, are equally confusing as concerns the definition of HR programmes in the Czech Republic. Apparently, the law providing for the portfolio of the Ministry of Labour and Social Affairs disregards the entire health component or any references thereto, in spite of the fact that the authors of the stipulations have declared their efforts to specify the activities pursued by the programmes as a whole. In this respect, the Health Ministry-related legislation is even more vague and incomplete and as a result of the legislative changes since 2017, specific harm reduction services are not anchored in the health sector. Harm reduction is mentioned as part of professional care, without any further description.

The key to the understanding of the core of the problem may be looked for in the years 2009 and 2010, when the consequences of the global financial crisis also became fully manifested in the Czech and Slovak Republics. The lack of financial resources exacerbated the hitherto little-discussed disagreement about the process of the integration of harm reduction services into the system of healthcare. These services, in fact, were the first in line to fall victim to the situation in the Czech Republic by losing almost the whole of their previous financial support from the health portfolio. Simply speaking, these two years, for the first time, displayed the consequences of the above-described processes in concrete terms. While the Ministry of Labour and Social Affairs maintained a rising tendency in the funding of social services, including those for people who use drugs, until 2009, the Ministry of Health recorded long-term cuts in funding which led to a decrease in financial support for HR programmes. In addition, the year 2010 experienced a drop in resources provided from all the sources of funding available (Table 3).

**Table 3:** Developments in funding from the national budgets in the Czech Republic (2005–2018, in thousands of EUR)

Source	2005	2006	2007	2008	2009	2010	2011	2012	2013	2014	2015	2016	2017	2018
ADF/Subvention from Government office in total (CR)	3547	3838	3762	4008	3686	3381	3695	3599	3690	3385	3482	4659	5428	7362
Harm reduction	n.a	n.a	1765	1952	1840	1744	1950	1911	1887	1804	1789	2194	2267	3064
MoH in total (? in CR)	1124	635	801	757	569	849	861	746	570	857	918	777	1368	1681
Harm reduction	n.a	n.a	105	159	163	64	79	150	195	156	293	367	503	739
MoLSA (? in CR)	1546	1753	2054	3186	3282	3628	3129	3355	3713	5195	5889	6857	7870	11,371
Harm reduction	n.a	n.a	1193	1808	1839	2013	1697	1809	2046	2342	2623	3241	3481	4524
HR Total in CR	n.a	n.a	3063	3919	3842	3821	3726	3870	4128	4302	4705	5801	6252	8327

Developments in the funding of drug services in total and HR services specifically provided from the respective national budgets. Analysis of financial support provided from the respective budgets of the Government Council for Drug Policy Coordination (Government office), Ministry of Health (MoH), and Ministry of Labour and Social Affairs (MoLSA)

Average exchange rates in the respective years were used for the re-calculation of expenses from CZK to EUR

## Discussion

Compelling evidence from across the world shows that harm reduction interventions are cost-effective; NSPs are one of the most cost-effective public health interventions in existence and can be cost-saving in the long term. There is evidence that a decrease in, or total cessation of, harm reduction services can lead to a spike in HIV and/or HCV infections. However, the harm reduction funding is in crisis and only few governments are investing substantially in harm reduction, even where the need is great [64, 65].

The key information implied by the trends indicated above is the relative proportions of all the main sources and their developments. The tendency is evident: while the social component of financial support shows a high degree of stability, and even a slight increase until 2010, in the Czech Republic, the health component managed by the Czech Ministry of Health constitutes only a fraction of the financial resources earmarked for harm reduction. Initially, the Ministry of Health even refused to cover the health component of these programmes in 2010 at all. In Slovakia, it is not possible to fund HR services using subsidies from the Slovak Ministry of Health, which results in the HR programmes and their health component being provided for financially even less than is the case in the Czech Republic. Thus, for Slovakia, the year 2010 meant a major threat to the system of HR services, which did not rest on very solid foundations even before then. It is apparent that the insufficient (mainly in legislative terms) integration of these services into the healthcare system is currently responsible for these services being those most affected by the situation resulting from the overall decrease in the health sector’s budget for the drug policy.

This situation further illustrates that the health component of harm reduction programmes was never appropriately integrated into the broader framework of health services. As a result, it relies fully on the system of subsidies, which are currently very uncertain (regional sources and alternative means of funding are not part of this analysis). Additionally, the financing system for short-term subsidies is multi-source, with each donor having their limitations regarding what the money can be spent on (e.g. salaries, injecting paraphernalia). These limitations contribute to uncertainty, and the rigidity of the funding rules does not allow the dynamically changing circumstances and evolving needs of clients to be responded to [55]. Harm reduction is historically one of the four cornerstones of the Czech drug policy [66, 67], and since 2019 even part of the main goal of the Czech strategy [68] and one of the five priorities of the Slovak strategy [69, 70]. Despite the stable position of harm reduction in the strategic documents of both countries, it has become clear that it has not been appropriately incorporated into the relevant systems in either country.

In Slovakia, neither the National Anti-Drug Strategy 2013–2020 nor the two consecutive Action Plans have associated budgets, nor have the necessary funding to implement activities been quantified. The present-day one-sided preference for a more feasible option of funding harm reduction as social services causes a serious imbalance and poses a threat to the underlying public health aspect of harm reduction services. The health component of HR programmes has been underfunded in the long term. Until recently, this shortage of funds was compensated for by using other sources. (In the Czech Republic, a greater proportion of support was previously provided from the budget of the Government Council for Drug Policy Coordination, while in the Slovak Republic, financial support was sought from OSF sources and the Anti-drug Fund provided a certain contribution^6^.) However, these alternative sources of funding have become unavailable, or their availability is uncertain. The problem must be addressed at the governmental level, particularly within the health portfolio, as a structural issue. The Czech Government Council for Drug Policy Coordination, previously a significant source of funding for HR services, has become unexpectedly insecure because of cuts in its budget following the financial crisis in 2008. Moreover, it tends to be affected by political twists and upheavals associated with changes of governments and their priorities, as has been experienced several times in the past [29]. However, it should be noted that despite its structural problems, the existence of an inter-disciplinary and inter-ministerial funding scheme managed by the Government Council for Drug Policy Coordination and its secretariat located in the Office of the Government makes a difference and provides substantial long-term support for harm reduction services for people who use drugs; this is also demonstrated when comparing the funds available for HIV prevention in PWID and MSM [71].

The Slovak system does not provide structural and sustainable support for HR programmes at all. Its overall financial instability and vulnerability is demonstrated by the consequences of the loss of support from the OSF. The withdrawal of this previously steady source of income resulted in reductions in the system of the order of hundreds of thousands of Euros.^7^ For Slovakia, thus, the year 2010 involved the risk of the destruction of the last remains of the network of services, which did not rest on solid foundations even before then. This instability has lately been manifested at all levels of the provision for the health component of harm reduction programmes, particularly needle and syringe exchange programmes (which is particularly alarming and, at the same time, illustrative) (Table 4). The Slovak funding system remains unstable despite the fact that a cost–benefit analysis of one of the harm reduction programmes in Slovakia found that every Euro invested in harm reduction generated benefits worth three Euros [75].

**Table 4:** Developments in funding from the national budgets in the Slovak Republic (2005–2018, in thousands of EUR)

Source	2005	2006	2007	2008	2009	2010	2011	2012	2013	2014	2015	2016	2017	2018
ADF/Subvention from Government office in total (SR)*	1656	1928	1314	407	1461	639	500	484	–	–	–	–	–	–
Harm reduction	88	349	66	81	54	120	62	55	–	–	–	–	–	–
MoH in total (? in SR)	1124	635	801	757	569	849	861	n.a	515	515	515	510	538	520
Harm reduction	n.a	n.a	105	159	163	64	79	n.a	33	30	50	41	53	40
MoLSA (? in SR)	1546	1753	2054	3186	3282	3628	3129	3087	2791	2709	2910	2910	n.a	3025
Harm reduction	n.a	n.a	1193	1808	1839	2013	1697	57	33	32	17	36	29	30
OSF (N/A in SR) HR**	54	21	–	–	–	–	–	–	–	–	–	–	–	–
HR Total in SR	142	370	1364	2048	2056	2197	1838	112	66	62	67	77	82	70

Developments in the funding of drug services in total and HR services specifically provided from the respective national budgets. Analysis of financial support provided from the respective budgets of the Anti-Drug Fund (ADF), Open Society Foundation (OSF), Ministry of Health (MoH), and Ministry of Labour and Social Affairs (MoLSA)

^*^Till May 2009 ADF; 2009–2012 Subvention from Government Office; from 2013 MoH

^**^In 2001 and 2002, for example, support amounted to approximately EUR 144 and EUR 121 thousand, respectively. In the following years, funding was reduced, and in the years 2005 and 2006 both the programmes and the support were brought to an end

As suggested above, both in the Czech Republic and in Slovakia, harm reduction services are gradually being transformed into social services. This results from the pressure exerted by the Ministry of Labour and Social Affairs as an increasingly stronger and predominant donor of these services, as the MoLSA succeeded in making HR services an integral part of social services by incorporating them into the broader framework of social services. A social worker, the position defined in the Act on Social Services, with all the related procedures and particulars (career development system, terms of operation for social services, and other legislative rules) being guaranteed, became the relevant professional for this segment. HR services are becoming increasingly adjusted to the standards of social services and losing confidence in other sources of funding. As the Ministry of Labour and Social Affairs has paid relatively willingly for what are denominated and categorised as social services, less and less emphasis is being placed on the core of such services, i.e. public health interventions. Traditionally, the ministries of health in both countries have for a long time adopted a very reserved, nowadays even negative, standpoint on the financial coverage of the health segment of these programmes. Not being embedded in the healthcare portfolio, HR services do not have their relevant position of a health professional defined and the service is not provided for in the legislation, including all the related procedures and particulars, as is the case in the portfolio managed by the MoLSA. The instability or absence of funding of HR services by the health portfolio makes their sustainability uncertain. The existence of HR services, including their health component, depends on sources other than the Ministry of Health. This makes the entire network structurally vulnerable, which was proved in practice in 2010. In Slovakia, following the termination of the financial support from the OSF and the concurrent failure of the Anti-Drug Fund, the system of HR services almost collapsed. In the Czech Republic, as a result of the absence of support from the Ministry of Health and severe cuts in the budget of the Government Council for Drug Policy Coordination, the programmes’ health component has also been under great pressure. Should the GCDPC fail in any way as a provider of support, the entire system may collapse. Unlike in Slovakia, however, the network would survive, maintaining only its social service aspect, while the health component would be destroyed. Given the circumstances, however, is it still possible to refer to such services as harm reduction? Such programmes would hardly fulfil their basic mission, i.e. to protect the health of individuals and the community. On the other hand, apart from concerns, a positive element can be found in that situation: it has been demonstrated that even in times of financial crisis these services are firmly embedded at least in the social portfolio, which provides a significant sense of support and elementary stability in the entire field. The advantage of multiple-source funding was also clearly shown; the breakdown of one source of income does not necessarily mean the total destruction of the system.

## Conclusions

As a conclusion, we postulate that: (a) there is a lack of systemic grounding of harm reduction services as interdisciplinary health-social services, which makes them difficult to operate within standard legislative and financial frameworks; (b) the sustainable development of harm reduction services is impossible without specific inter-disciplinary and inter-ministerial support and a policy accent; (c) harm reduction was introduced through grassroots civil society initiatives and they broadly remain associated with the NGO sector. Thus, the involvement of civil society in drug policy coordination mechanisms is also key for the implementation of harm reduction programmes; (d) the existence of a functional strategic framework and funding schemes in drug policy, including harm reduction as priority measures, provides an essential basis for the development and sustainability of harm reduction measures, and (e) an ideology-driven versus an evidence-based approach to drug policy complicates the implementation and support of harm reduction services.

## Data Availability

Data sharing is not applicable to this article as no datasets were generated or analysed during the current study. Please contact author for data requests for data cited in the article.

## Footnotes

1 Out of all the Central European countries mentioned above, the Czech Republic, for example, reports by far the largest numbers of syringes exchanged and the widest coverage by opiate substitution treatment [14], although it ranks among the countries with a very low prevalence of HIV among PWID. In addition, it also began surveying and strengthening the segment of providing sterile injecting paraphernalia via pharmacies [27, 28].

2 The national estimates suggest that there were approximately 41.7 thousand “problem drug users” (central estimates) in the Czech Republic in 2017 and 18.8 thousand in Slovakia in 2006 [35, 36].

3 Note, for example, the initiation of the first study in the Czech Republic to use the Rapid Assessment and Response (WHO) methodology conducted in 1995 [59].

4 As part of the IGA NR9447 project, a proposal for more intensive cooperation with pharmacies on harm reduction interventions was drafted; it is estimated that pharmacies sold 1.5 million syringes to PWID in 2009 [27, 28].

5 This is in contradiction of the integrative approach to such services as recommended by the key EU documents cited in the introductory section above.

6 In 2008, for example, when considering grant applications concerning support for needle and syringe exchange projects, the Slovak General Secretariat of the Ministerial Committee for Drug Addiction and Drug Control strictly required the providers to be accredited by the Ministry of Labour, Social Affairs, and the Family according to Act No. 5/2005 on social and legal protection and social guardianship adopted by the National Council of the Slovak Republic. In other words, it demanded that programmes such as those providing needle and syringe exchange (if they wanted to receive financial support) be accredited and present themselves as social services. Paradoxically, the above-cited Bulletin of the Ministry of Health of the Slovak Republic, Sect. 12–15, provides for HR services and assigns needle and syringe exchange interventions (including those provided in non-governmental facilities) to health professionals.

7 For example, EUR 144 thousand in 2001, EUR 120 thousand in 2002, and EUR 64 thousand in 2003 [72–74]. In 2008 and 2009, moreover, what is called the governmental Anti-drug Fund provided only minimal support for these services.

## References

[1] Grund JP, Breeksema J, Colson R, Bergeron H (2017). Drug Policy in The Netherlands. European drug policies: the ways of reform.

[2] Hedrich D, Rhodes T (2010). Harm reduction: evidence, impacts and challenges. European Monitoring Centre for Drugs and Drug Addiction (EMCDDA).

[3] Harm Reduction International. Global State of Harm Reduction 2018. https://www.hri.global/files/2018/12/11/global-state-harm-reduction-2018.pdf. Accessed 15 Apr 2020.

[4] Des Jarlais D, Guardino V, Arasteh K, McKnight C, Milliken J, Purchase D. Current State of Syringe Exchange Programs in the US: Crisis and Opportunity, 2010.https://www.researchgate.net/publication/266901146_Current_State_of_Syringe_Exchange_Programs_in_the_US_Crisis_and_Opportunity. Accessed 15 Apr 2020.

[5] Grund JP (2009). Harm reduction in the United States at a moment of change: moving innovation from grassroots to mainstream?. Addiction.

[6] IHRA (2009). What is Harm Reduction? A position statement from the International Harm Reduction Association.

[7] Advisory Council on the Misuse of Drugs (1988). AIDS and drug misuse Pt 1.

[8] WHO (1986). Drug dependence and alcohol-related problems.

[9] Rhodes T (2002). The 'risk environment': a framework for understanding and reducing drug-related harm. Int J Drug Policy.

[10] Rhodes T (2009). Risk environments and drug harms: a social science for harm reduction approach. Int J Drug Policy.

[11] Wodak A (2009). Harm reduction is now the mainstream global drug policy. Addiction.

[12] WHO, UNODC, UNAIDS. Technical Guide for countries to set targets for universal access to HIV prevention, treatment and care for injecting drug users. 2009. www.who.int/hiv/pub/idu/targetsetting/en/index.html. Accessed 15 Apr 2020.

[13] European Monitoring Centre for Drugs and Drug Addiction (EMCDDA). Perspectives on drugs. Drug consumption rooms: an overview of provision and evidence. Lisbon: EMCDDA; 2018. https://www.emcdda.europa.eu/topics/pods/drug-consumption-rooms_en. Accessed 15 Apr 2020.

[14] European Monitoring Centre for Drugs and Drug Addiction (EMCDDA). European Drug Report 2019: Trends and Developments. Luxembourg: Publications Office of the European Union; 2019.

[15] European Monitoring Centre for Drugs and Drug Addiction (EMCDDA). Health and social responses to drug problems: a European guide. Luxembourg: Publications Office of the European Union; 2017.

[16] Sporer KA, Kral AH (2007). Prescription naloxone: a novel approach to heroin overdose prevention. Ann Emerg Med.

[17] European Monitoring Centre for Drugs and Drug Addiction (EMCDDA). Perspectives on drugs. Preventing overdose deaths in Europe. Lisbon: EMCDDA; 2018. https://www.emcdda.europa.eu/topics/pods/preventing-overdose-deaths

[18] Grund JP, Coffin P, Jauffret-Roustide M, de Bruin D, Dijkstra M, Blanken P. The fast and furious: cocaine, amphetamines and harm reduction. In: European Monitoring Centre for Drugs and Drug Addiction (EMCDDA)—Rhodes T, Hedrich D, editors. Harm reduction: evidence, impacts and challenges. Luxembourg: Publications Office of the European Union; 2010, p. 205–254.

[19] Wiessing L, Likatavicius G, Klempová D, Hedrich D, Nardone A, Griffiths P (2009). Associations between availability and coverage of HIV-prevention measures and subsequent incidence of diagnosed HIV infection among injection drug users. Am J Public Health.

[20] Craine N, Hickman M, Parry JV (2009). Incidence of hepatitis C in drug injectors: the role of homelessness, opiate substitution treatment, equipment sharing, and community size. Epidemiol Infect.

[21] Van den Berg C, Smit C, Van Brussel G, Coutinho RA, Prins M (2007). Full participation in harm reduction programmes is associated with decreased risk for human immunodeficiency virus and hepatitis C virus: evidence from the Amsterdam cohort studies among drug users. Addiction.

[22] Kimber J, Palmateer N, Hutchinson S, Hickman M, Goldberg D, Rhodes T. Harm reduction among injecting drug users—evidence of effectiveness. In: European Monitoring Centre for Drugs and Drug Addiction (EMCDDA)—Rhodes T, Hedrich D, editors. Harm reduction: evidence, impacts and challenges. Luxembourg: Publications Office of the European Union; 2010.

[23] European Monitoring Centre for Drugs and Drug Addiction (EMCDDA). Annual report 2010: the state of the drugs problem in Europe. Luxembourg: Publications Office of the European Union; 2010.

[24] Grund JP, Zabransky T, Irwin K, Heimer R, Pates R, Riley D (2009). Stimulant use in Central & Eastern Europe: how recent social history shaped current drug consumption patterns. Interventions for amphetamine misuse.

[25] Van Hout MC (2014). Kitchen chemistry: a scoping review of the diversionary use of pharmaceuticals for non-medicinal use and home production of drug solutions. Drug Test Anal.

[26] European Monitoring Centre for Drugs and Drug Addiction (EMCDDA) (2002). Report on the drug situation in the Candidate CEECs.

[27] Gabrhelík R, Miovská L, Miovský M (2008). Dotazníkový průzkum harm reduction intervencí v lékárnách České republiky [Questionnaire Survey of Harm Reduction Interventions in Czech Pharmacies]. Alkoholizmus a drogové závislosti.

[28] Gabrhelík R, Miovský M (2009). Možnosti a meze současného využití sítě lékáren z hlediska jejich participace na nízkoprahových veřejnozdravotních intervencích v rámci adiktologických služeb [The Use of the Current Network of Pharmacies for Low-Threshold Public Health Interventions as Part of Addiction Treatment Services: Possible Benefits and Limitations]. Adiktologie.

[29] Radimecký J (2007). Rhetoric versus practice in Czech drug policy. J Drug Issues.

[30] Miovský M (2007). Changing patterns of drug use in the Czech Republic during the post-communist era: a qualitative study. J Drug Issues.

[31] Grund JP, Pates R, McBride A, Arnold K (2005). The eye of the needle: an ethno-epidemiological analysis of injecting drug use. Injecting Illicit drugs.

[32] Miles MB, Huberman AM (1994). Qualitative data analysis: an expanded sourcebook.

[33] Čermák I, Štěpaníková I (1998). Kontrola validity dat v kvalitativním výzkumu [Checking data validity in qualitative research]. Československá psychologie.

[34] Miovský M (2006). Kvalitativní přístup a metody v psychologickém výzkumu [A Qualitative Approach and Methods in Psychological Research].

[35] Mravčík V, Chomynová P, Grohmannová K, Janíková B, Černíková T, Rous Z, Tion Leštinová Z, Nechanská B, Cibulka J, Fidesová H, Vopravil J. Výroční zpráva o stavu ve věcech drog v České republice v roce 2018 [Annual Report on the Drug Situation 2018—Czech Republic]. Mravčík V. (ed.). Prague: Office of the Government of the Czech Republic; 2019.

[36] European Monitoring Centre for Drugs and Drug Addiction (EMCDDA). Statistical bulletin 2010, Table PDU-1, Part (ii) Injecting drug use. Lisbon: EMCDDA; 2010. https://www.emcdda.europa.eu/stats10/pdutab1b. 14 Feb 2011.

[37] Mravčík V, Chomynová P, Grohmannová K, Nečas V, Grolmusová L, Kiššová L, Nechanská B, Sopko B, Fidesová H, Vopravil J, Jurystová L. Výroční zpráva o stavu ve věcech drog v České republice v roce 2013 [Annual Report on the Drug Situation 2013—Czech Republic]. Mravčík V. (ed.). Prague: Office of the Government of the Czech Republic; 2014.

[38] European Monitoring Centre for Drugs and Drug Addiction (EMCDDA). Slovakia, Country Drug Report 2017. Luxembourg: Publications Office of the European Union; 2017. https://www.emcdda.europa.eu/system/files/publications/4506/TD0116920ENN.pdf. Accessed 23 Apr 2020.

[39] European Monitoring Centre for Drugs and Drug Addiction (EMCDDA). Slovakia, Country Drug Report 2018. Luxembourg: Publications Office of the European Union; 2018. https://www.emcdda.europa.eu/publications/country-drug-reports/2018/slovakia_en. Accessed 23 Apr 2020.

[40] European Monitoring Centre for Drugs and Drug Addiction (EMCDDA). Slovakia, Country Drug Report 2019. Luxembourg: Publications Office of the European Union; 2019. https://www.emcdda.europa.eu/publications/country-drug-reports/2019/slovakia_it. Accessed 23 Apr 2020.

[41] NMCD. Stav drogových závislostí a kontrola drog v Slovenskej republike [Drug addiction and drug control in the Slovak Republic], Národné monitorovacie centrum pre drogy. Bratislava: Úrad vlády SR; 2006.

[42] NMCD. Výročná správa o stave drogovej problematiky na Slovensku v roku 2006 [National Report on the Drug Situation 2006—Slovakia], Národné monitorovacie centrum pre drogy. Bratislava: Úrad vlády SR; 2007.

[43] NMCD. Výročná správa o stave drogovej problematiky na Slovensku za rok 2007 [National Report on the Drug Situation 2007—Slovakia], Národné monitorovacie centrum pre drogy. Bratislava: Úrad vlády SR; 2008.

[44] NMCD. Výročná správa o stave drogovej problematiky na Slovensku za rok 2008 [National Report on the Drug Situation 2008—Slovakia], Národné monitorovacie centrum pre drogy. Bratislava: Úrad vlády SR; 2009.

[45] NMCD. Slovakia: New development, Trends and In-depth Information on Selected Issues. 2010 National Report (2009 data) to the EMCDDA by the Reitox National Focal Point., Národné monitorovacie centrum pre drogy. Bratislava: Úrad vlády SR; 2010.

[46] NMCD. Prehľad vybraných údajov o stave drogovej problematiky na Slovensku v roku 2010 [Drug Situation in Slovakia in 2010—an overview of selected data]. Bratislava: Národné monitorovacie centrum pre drogy; 2011.

[47] NMCD. Slovakia: New development, Trends and In-depth Information on Selected Issues. 2011 National Report (2010 data) to the EMCDDA by the Reitox National Focal Point. Národné monitorovacie centrum pre drogy. Bratislava: Úrad vlády SR; 2011.

[48] NMCD. Prehlad vybraných údajov o stave drogovej problematiky na Slovensku v roku 2011 [Drug Situation in Slovakia in 2011—an overview of selected data]. Bratislava: Národné monitorovacie centrum pre drogy; 2012.

[49] NMCD. Stav drogovej problematiky na Slovensku: Súhrn Výročnej správy NMCD o stave drogovej problematiky v roku 2011 [Drug Situation in Slovakia: a summary of the National Report on the Drug Situation 2011—Slovakia published by the National Monitoring Centre for Drugs]. Bratislava: Národné monitorovacie centrum pre drogy; 2012.

[50] NMCD. Stav drogovej problematiky na Slovensku: Súhrn Výročnej správy NMCD o stave drogovej problematiky v roku 2012 [Drug Situation in Slovakia: a summary of the National Report on the Drug Situation 2012—Slovakia published by the National Monitoring Centre for Drugs], Národné monitorovacie centrum pre drogy. Bratislava: Ministerstvo zdravotníctva SR; 2013.

[51] NMCD. Stav drogovej problematiky na Slovensku: Súhrn Výročnej správy NMCD o stave drogovej problematiky v roku 2013 [Drug Situation in Slovakia: a summary of the National Report on the Drug Situation 2013—Slovakia published by the National Monitoring Centre for Drugs], Národné monitorovacie centrum pre drogy. Bratislava: Ministerstvo zdravotníctva SR; 2014.

[52] NMCD. Stav drogovej problematiky na Slovensku: Súhrn Výročnej správy NMCD o stave drogovej problematiky v roku 2014 [Drug Situation in Slovakia: a summary of the National Report on the Drug Situation 2014—Slovakia published by the National Monitoring Centre for Drugs], Národné monitorovacie centrum pre drogy. Bratislava: Ministerstvo zdravotníctva SR; 2015.

[53] NMCD. Stav drogovej problematiky na Slovensku: Súhrn Výročnej správy NMCD o stave drogovej problematiky v roku 2015 [Drug Situation in Slovakia: a summary of the National Report on the Drug Situation 2015—Slovakia published by the National Monitoring Centre for Drugs], Národné monitorovacie centrum pre drogy. Bratislava: Ministerstvo zdravotníctva SR; 2016.

[54] Úrad verejného zdravotníctva Slovenskej republiky. Výskyt HIV infekcie v Slovenskej republike k 31.12.2018 [HIV infection in the Slovak Republic as of 31 December 2018]. 2019. https://www.uvzsr.sk/index.php?option=com_content&view=article&id=3765:vyskyt-hiv-infekcie-v-slovenskej-republike-k-31122018&catid=68:epidemiologia&Itemid=76

[55] Kender-Jeziorska I (2019). Needle exchange programmes in Visegrad countries: a comparative case study of structural factors in effective service delivery. Harm Reduct J.

[56] Fric P, Bútora M. The role of the nonprofit sector in public policy In: Potůček M, LeLoup LT, Jenei G, Váradi L, editors. Public policy in Central and Eastern Europe: theories, methods, practices. Bratislava: NISPAcee; 2003.

[57] Ministerstvo zdravotníctva SR. Vestník MZ SR 2003, čiastka 12-15 [Bulletin of the Slovak Health Ministry, 2003, Section 12-15]. Bratislava: Ministerstvo zdravotníctva SR; 2003.

[58] Kalina K (2007). Developing the System of Drug Services in the Czech Republic. J Drug Issues.

[59] Tyrlik M, Zuda T, Bem P, Power R (1996). Rapid assessment of the drug abuse situation in the Czech Republic. Bull Narc.

[60] Mravčík V, Škařupová K, Orlíková B, Zábranský T, Karachaliou K, Schulte B (2011). Use of gelatine capsules for application of methamphetamine: a new harm reduction approach. Int J Drug Policy.

[61] Guryčová Z (2010). Perorální aplikace pervitinu formou želatinové kapsle [Oral Application of Pervitin with a Gelatine Capsule]. Adiktologie.

[62] Nadácia otvorenej spoločnosti. Minimálne Štandardy nízkoprahových služieb pre užívateľov/užívateľky drog v zmysle Harm Reduction [Harm Reduction-oriented Minimum Standards for Low-threshold Drug Services]. A working version of the standards developed as part of the “Outreach Work Standards” project carried out by the STORM civic association affiliated with Constantine the Philosopher University in Nitra from 2005 to 2006, with support from NOS-OSF. Bratislava: NOS-OSF; 2005.

[63] Okruhlica L. Posudok: Minimálne Štandardy nízkoprahových služieb pre užívateľov/užívateľky drog v zmysle Harm Reduction [Harm Reduction-oriented Minimum Standards for Low-threshold Drug Services: an expert evaluation]. An expert evaluation of the working version of the standards completed on 8 April 2007. Bratislava; 2007.

[64] HRI. Making the investment case: Cost-effectiveness evidence for harm reduction. London: Harm Reduction International. 2020. https://www.hri.global/contents/2027

[65] Cook C, Davies C (2018). The Lost Decade: Neglect for harm reduction funding and the health crisis among people who use drugs.

[66] Secretariat of the Government Council for Drug Policy Coordination. National Drug Policy Strategy for the Period 2005–2009. Prague: Office of the Government of the Czech Republic; 2004.

[67] Secretariat of the Government Council for Drug Policy Coordination. National Drug Policy Strategy for the Period 2010–2018. Prague: Office of the Government of the Czech Republic; 2010.

[68] Secretariat of the Government Council for Drug Policy Coordination. National Strategy to Prevent and Reduce the Harm Associated with Addictive Behaviour 2019–2027. Prague: Office of the Government of the Czech Republic; 2019.

[69] Government of the Slovak Republic. National Anti-Drug Strategy for the Period 2009–2012. Bratislava: Government of the Slovak Republic; 2009. https://www.emcdda.europa.eu/attachements.cfm/att_35509_EN_Slovak%20Republic%20Strategy%2020

[70] Ministry of Health of the Slovak Republic. National Antidrug Strategy of the Slovak Republic 2013–2020. Bratislava: Ministry of Health of the Slovak Republic; 2013. https://www.emcdda.europa.eu/drugs-library/national-antidrug-strategy-slovak-republic-2013%e2%80%932020_en

[71] Mravčík V, Pitoňák M, Hejzák R, Janíková B, Procházka I (2018). HIV/AIDS epidemic in the Czech Republic and related factors: comparison of key populations of people who inject drugs and men who have sex with men. Adiktologie.

[72] Nadácia otvorenej spoločnosti. Výročná správa Nadácie otvorenej spoločnosti 2001 [Open Society Foundation – 2001 Annual Report], Bratislava: NOS-OSF; 2002.

[73] Nadácia otvorenej spoločnosti. Výročná správa Nadácie otvorenej spoločnosti 2002 [Open Society Foundation – 2002 Annual Report], Bratislava: NOS-OSF; 2003.

[74] Nadácia otvorenej spoločnosti. Výročná správa Nadácie otvorenej spoločnosti 2003 [Open Society Foundation – 2003 Annual Report], Bratislava: NOS-OSF; 2004.

[75] Filko M, Hojčková M, Macha J, Melioris L. Program Chráň sa sám sa zaplatí trojnásobne: Analýza nákladov a prínosov programu výmeny injekčných striekačiek OZ Odyseus. Inštitút finančnej politiky [The Protect Yourself Programme will pay for itself three times over: cost-benefit analysis of the needle exchange programme run by the Odyseus association.] Inštitút finančnej politiky. 2015. https://www.mfsr.sk/sk/financie/institut-financnej-politiky/publikacie-ifp/komentare/15-program-chran-sam-zaplati-trojnasobne-jun-2015.html

